# Severe Eosinophilia in a Case of Giardiasis

**DOI:** 10.4084/MJHID.2011.009

**Published:** 2011-03-17

**Authors:** Rifat Nadeem Ahmad, Ahmed Sherjil, Asad Mahmood, Shahid Rafi

**Affiliations:** 1 Department of Pathology and; 2 Department of Pediatrics, Heavy Industries Taxila Hospital, Taxila, Pakistan; 3 Department of Pathology, Armed Forces Institute of Cardiology, Rawalpindi, Pakistan.”; 4 Department of Pathology, Shifa College of Medicine, Islamabad, Pakistan

**Dear Editor,**

*Giardia lamblia* is a common intestinal parasite with worldwide distribution. Although primarily presenting with gastrointestinal complaints, the clinical manifestations of giardiasis can be varied. A number of patients are asymptomatic and fever is an uncommon finding.^1^ The infection is generally not associated with haematological abnormalities and the presence of eosinophilia is most unusual.^1^,^2^ An atypical presentation of giardiasis, when associated with rare findings such as eosinophilia can lead to diagnostic predicament or raise false alarms as happened in the case of a previously healthy nine years old boy, who presented in a secondary care hospital at Taxila in Northern Pakistan in April 2009. He had a four-day history of high grade fever and vague abdominal discomfort. There was no history of diarrhoea, constipation, vomiting or any drug intake. Systemic examination was unremarkable. He was admitted to the hospital and given symptomatic treatment pending results of investigations, which included complete blood counts and stool examination. Blood picture showed a total leucocyte count (TLC) of 86.6x10^9^/l with an absolute eosinophil count of 78.2x10^9^/l, while stool examination revealed semi-formed stools containing cysts of *Giardia lamblia*. The high TLC raised concerns about a myeloproliferative disorder. However, as no immature cells were seen on peripheral blood smear microscopy, it was decided to first treat giardiasis before considering bone marrow examination. The patient was administered oral metronidazole 15 mg/kg/day in three divided doses for seven days. Stool samples were examined on three consecutive days to exclude helminthic infestations. No antihelminthic preparations were given. The patient became afebrile on second day of hospitalization and his eosinophil count dropped to 39.8x10^9^/l on fifth day, when he was discharged with instructions to report back after seven days. On follow-up, he was symptom-free with an absolute eosinophil count of 16.8x10^9^/l on seventh day after discharge and 0.3x10^9^/l three weeks later.

Eosinophilia is defined as an absolute eosinophil count of >0.35x10^9^/l in peripheral blood and is considered severe if the count is >5x10^9^/l.^3^ Severe eosinophilia is associated with a number of conditions including helminthic infections, precursor B-cell acute lymphoblastic leukemia, precursor T-cell lymphoblastic lymphoma and various hypereosinophilic syndromes usually characterized by end-organ involvement.^4^ A few cases of giardiasis associated with eosinophilia have been reported^5^–^7^ but the extremely high eosinophil count seen in our patient is virtually unknown. The unusual presentation of giardiasis in this case could have posed a diagnostic dilemma. Fortunately, the positive stool examination for *Giardia lamblia* kept us on the right track. The prompt resolution of eosinophilia following treatment of giardiasis with metronidazole appears to confirm a causal relationship with the parasite. A similar reduction of blood eosinophilia following metronidazole therapy in a case of Churg-Strauss syndrome with giardiasis in Italy supports this association.^6^ However, the pathogenesis of eosinophilia in these cases is not known. The rarity of this finding implies that infection with *Giardia lamblia* alone is not enough to trigger an eosinophilic response and there must be other factors involved. Dos Santos and Vituri, who reported an association of eosinophilia with giardiasis in a Brazilian study, have suggested the possible role of *Giardia lamblia* allergens in provoking eosinophilia,^7^ while Ferrante et al have attributed this finding to an interaction between different eosinophilopoietic stimuli.^6^ However, these are conjectures and the exact mechanism remains unexplained. Contrary to the general perception that *Giardia lamblia* only causes gastrointestinal disease and that giardiasis does not lead to haematological changes; it not only presents in a variety of ways without diarrhoea but can also cause reactive eosinophilia in patients with sub-clinical infection. The possibility of giardiasis should be kept in mind while diagnosing a case of unexplained eosinophilia.
